# Development of a Peptide that Selectively Activates Protein Phosphatase-1 in Living Cells**

**DOI:** 10.1002/anie.201204308

**Published:** 2012-09-07

**Authors:** Jayanta Chatterjee, Monique Beullens, Rasa Sukackaite, Junbin Qian, Bart Lesage, Darren J Hart, Mathieu Bollen, Maja Köhn

**Affiliations:** Genome Biology UnitEMBL, Meyerhofstrasse 1, 69117 Heidelberg (Germany); Laboratory of Biosignaling & Therapeutics, Katholieke Universiteit LeuvenLeuven (Belgium); EMBL Grenoble Outstation and Unit of Virus Host-Cell InteractionsUMI3265 UFJ-EMBL-CNRS, Grenoble (France)

**Keywords:** cell-penetrating peptides, drug design, enzyme activation, protein phosphatase-1, protein–protein interactions

## Abstract

**The first cell-penetrating peptide:**

that activates protein phosphatase-1 (PP1) by disrupting a subset of PP1 complexes in living cells has been developed. Activated PP1 rapidly dephosphorylates its substrates, counteracting kinase activity inside cells. Activation of PP1 can thus be a novel approach to study PP1 function and to counteract Ser/Thr kinase activity under pathologically increased kinase signaling.

Ser/Thr phosphorylation in proteins is fundamental to signal transduction processes in cells. Malfunction of these processes contributes to the development and progress of diseases. The phosphorylation of proteins on Ser and Thr residues is mediated by protein Ser/Thr kinases, whereas protein Ser/Thr phosphatases (PSTPs) hydrolyze protein-bound phosphomonoesters. Inhibitors of Ser/Thr kinases are widely used for functional studies and as therapeutic agents, but there are virtually no tools to target specifically the counteracting PSTPs in living cells.^[1]^ The major obstacle for the design of selective inhibitors of PSTPs lies in the similarity of the catalytic pocket, which so far has not allowed the development of selective, high-affinity active-site binders. Therefore, widely used potent inhibitors of PSTPs are not very useful for functional studies.^[2]^ Alternatively, enzyme activators have recently gained much attention.^[3]^ Although a possible activator of the PSTP protein phosphatase-1 (PP1) was reported in vitro,^[4]^ no studies for potency and selectivity in living cells have been presented to date. Thus, there is an urgent need to develop potent and selective molecules to target specific PSTPs in living cells.^[1,2]^

PP1 is a ubiquitous enzyme that is predicted to catalyze the majority of Ser/Thr dephosphorylation in eukaryotic cells.^[5,^^[6]^ PP1 has a broad specificity but is restrained in vivo by numerous PP1-interacting proteins (PIPs) (roughly 200 in vertebrates) that form holoenzymes with the PP1 catalytic subunit and function, for example, as activity regulators or substrate-targeting proteins.^[6,^^[7]^ An attractive approach for the development of PP1-selective effectors is targeting specific interfaces between PP1 and PIPs and disrupting their interaction. One of these interfaces, which occurs in about 90 % of all validated PIPs, is referred to as the RVxF-type PP1-binding motif (single-letter amino acid code, x=any amino acid), which binds to a site on PP1 that is remote from the active site.^[8]^ Synthetic peptides that contain variants of the RVxF motif, but not the corresponding RAxA peptides, were reported to disrupt a subset of PIP–PP1 complexes in vitro.^[4,^^7,^^9]^ In general, disruption of protein–protein interactions by peptides is an appealing strategy to interrogate signaling pathways owing to their high affinity and specificity.^[10]^ Nevertheless, it needs to be considered that the application of peptides in living cells is often hampered by insufficient cell permeability and proteolytic instability.^[10]^

In our work we sought to develop a proteolytically stable, cell-permeable peptide that specifically disrupts PP1–PIP interactions and modulates PP1 signaling in living cells. As a lead PP1-disrupting peptide (PDP) we synthesized **PDP0** (Table ^1]^), comprising the RVxF motif (underlined) and flanking sequences of the nuclear inhibitor of PP1 (NIPP1).^[9]^ The peptide was tested in a competition assay, in which **PDP0**-mediated release of PP1 from its PIP Inhibitor 2 (I2) was measured. I2 binds to PP1 through an RVxF motif and inhibits PP1. The release results in the deinhibition of PP1, which is measured using a phosphatase activity assay. **PDP0** alleviated the inhibition of PP1 by I2 with an EC_50_ of 87±10 nm (Table 1 and Figure S1 in the Supporting Information). An alanine scan of **PDP0** revealed the importance of the basic N-terminal stretch and the two C-terminal isoleucines for the potent deinhibition of PP1 (Figure 1 and Figure S2). These results were confirmed by C- and N-terminal truncation scans. When multiple amino acids were replaced with alanine at the two RVxF-flanking serine positions and the C-terminal acidic EDDE sequence, the disruption efficacy was further increased (Figure S3). Collectively, these data enabled us to design the optimized peptide **PDP1** (Table 1 and Figures S1 and S3).

**Figure 1:**
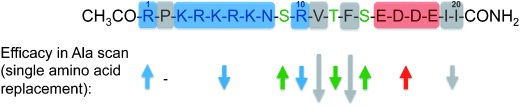
Schematic representation of the results of the alanine scan displaying relative changes in the efficacy of the peptides in disrupting PP1:I2 binding (see also Figure S2 in the Supporting Information). The upward arrow signifies an increase, the downward arrow a decrease in efficacy. Color legend: blue=basic, green=hydrophilic, gray=hydrophobic, red=acidic residues.

**Table 1:** Efficacy of the peptides in disrupting the PP1:I2 complex.[a]

Peptide	Sequence	EC_50_ [nm]
**PDP0**	RVTFSEDDEII	87±10
**PDP1**	RVTF**A**E**AA**EII	21±2
**PDP2**	RVTF**A**E**AA**EII	53±8
**PDP2m**	RATAAEAAEII	inactive
**PDP3**	RVTF*Bpa*E**AA**EII	176±13
**PDP3m**	RATA*Bpa*EAAEII	inactive

[a] See also Figure S1 in the Supporting Information. The results are presented as the mean±standard error of the mean (*n*=4). The efficacy of the peptides was determined by an in vitro phosphatase assay, measuring the activity of PP1 toward its ^32^P-labeled substrate glycogen phosphorylase *a*.^[9]^

As the RVxF motif of PIPs binds in an extended conformation to PP1,^[8]^ we sought to develop D-Pro-L-Pro-templated linear peptides (Figure S4 in the Supporting Information) and D-Pro-L-Dap (L-Dap=L-2,3-diaminopropionic acid) templated disulfide-bridged cyclic peptides to display the RVTF motif in an extended conformation (Scheme S1 and Figure S5).^[11]^ However, constraining the peptide by cyclization resulted in the loss of deinhibition potency, which is consistent with the required conformational flexibility of the RVxF motif and its flanking sequences for binding to PP1.^[6]^ Taken together, alanine-, truncation-, multiple-alanine-, and D-Pro-L-Pro/Dap-scans revealed that the basic amino acid stretch (-KRKRK-) N-terminal to the RVTF motif and two hydrophobic residues (-II-) C-terminal to the RVTF motif in **PDP0** are essential for binding to PP1. On the contrary, the acidic stretch (-EDDE-) and the two RVTF-flanking serines destabilize the binding of **PDP0** to PP1.

Next, we assessed the cell permeability of the peptides using confocal microscopy by incubating live cells with 5-carboxyfluorescein (FAM)-labeled peptides. Confocal microscopy was chosen because it can distinguish intracellular peptides from peptides adhering to the extracellular cell surface. FAM-**PDP1** did not penetrate cells efficiently (Figure 2 a), and showed extra- and intracellular aggregation in different cell types at higher concentrations (Figure S6 in the Supporting Information). We then applied an iterative design strategy involving the sequential addition of arginine/lysine residues to the N-terminus, thereby enhancing its cell penetration without significantly compromising its efficacy as a PP1–PIP-disrupting peptide. This led to the design of **PDP2**, which also disrupted the PP1:I2 complex but was slightly less efficient than the nonpermeable peptide **PDP1** (Table 1). The corresponding RATA mutant of **PDP2** (**PDP2m**), showed no effect in the deinhibition assays (Table 1), confirming the importance of valine and phenylalanine in mediating the high-affinity binding of **PDP2** to PP1.

**Figure 2:**
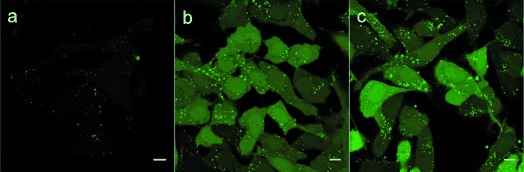
Cellular uptake of peptides illustrated by confocal images of live U2OS cells (human osteosarcoma cells) incubated with 25 μm of fluorescein-labeled PDP1 (a), PDP2 (b), and PDP2m (c) for 2 h. Scale bars represent 10 μm.

FAM-**PDP2** was efficiently taken up into different cell lines at different concentrations, showing a diffuse cytoplasmic and nucleoplasmic distribution, and also to a minor extent potential vesicular localization or aggregation (Figure 2 b, Figure S6 in the Supporting Information). The differences in the cell-penetration efficiency of **PDP1** and **PDP2** can be ruled to be independent of the FAM label, as both peptides carry it. The uptake of FAM-**PDP2** was lower at 4 °C than at 37 °C (Figure S7), suggesting both energy-dependent and -independent modes of uptake.^[12]^ FAM-**PDP2m** was taken up at least as well as FAM-**PDP2** (Figure 2 b,c).

Once cell penetration was established, we addressed the selectivity of **PDP2** toward binding the closely related phosphatases PP1 and PP2A. For this we precipitated biotinylated **PDP2** and **PDP2m** from HEK293 (human embryonic kidney cells) (Figure 3 a) and U2OS cell lysates (Figure S8 in the Supporting Information) with streptavidin-coated Sepharose beads, which resulted in a dose-dependent co-sedimentation of PP1. **PDP2** did not co-precipitate the structurally closest related PP2A (Figure 3 b), and did not show any specificity towards the three PP1 isoforms (α, β, and γ) (Figure S9). **PDP2m** co-precipitated neither PP1 nor PP2A (Figure 3 a,b). Furthermore, the efficacy of **PDP2** in disrupting PP1 holoenzyme complexes was analyzed in vitro with freshly immunoprecipitated PP1 complexes using PIP-directed antibodies. The complexes were subjected to **PDP2** and **PDP2m**, and subsequently assayed using ^32^P-labeled phosphorylase *a* as substrate, as detailed above. The resulting PP1 activity correlates with the level of released active PP1. The results demonstrate that **PDP2**, but not **PDP2m**, disrupted all the tested PP1 holoenzymes in vitro, further corroborating the potential of **PDP2** as a cell-permeable disruptor of PP1 holoenzymes.

**Figure 3:**
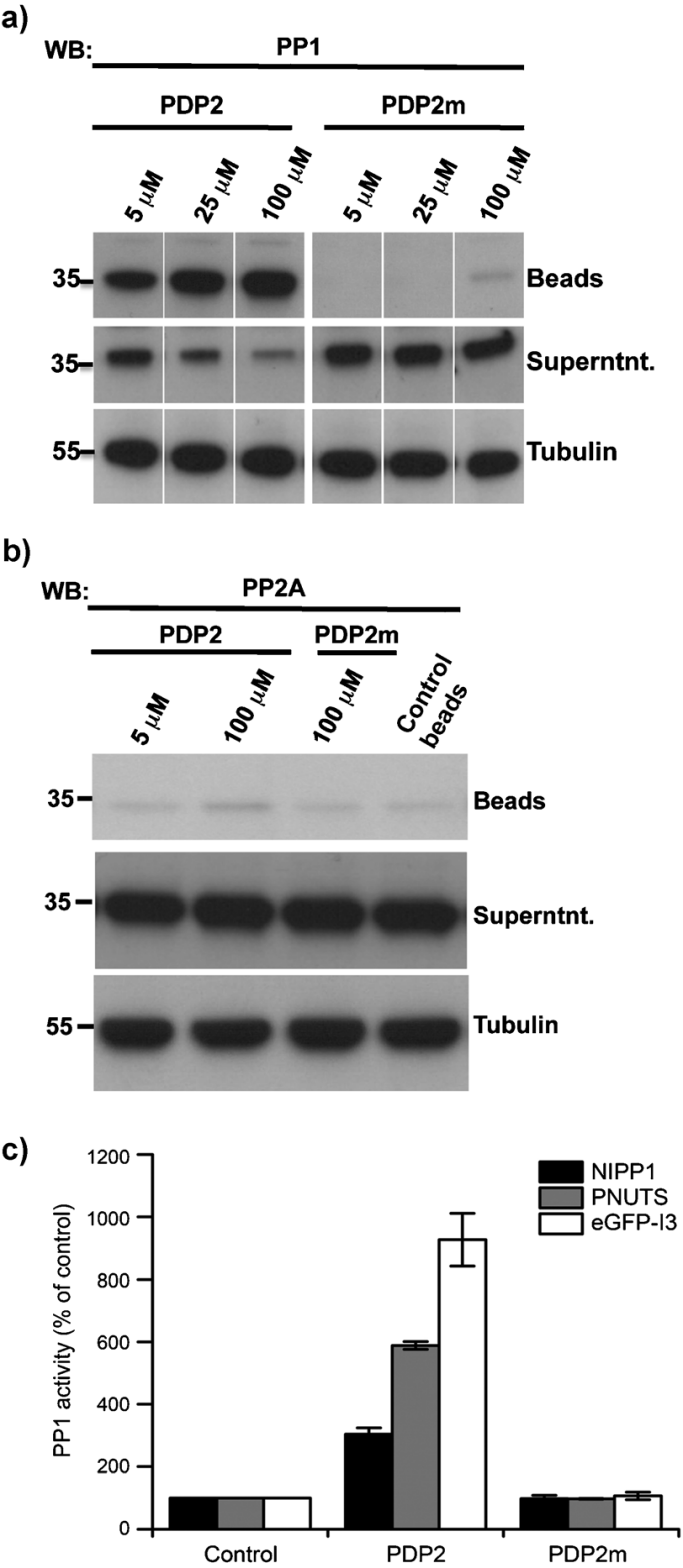
Selectivity and efficacy of PDP2 in disrupting the PIP:PP1 complex. a) Immunoblot of PP1 in a streptavidin pull-down of biotinylated PDP2 and PDP2m from HEK293 cell lysates. The full blot is shown in Figure S8 in the Supporting Information. b) Immunoblot of PP2A in a streptavidin pull-down of biotinylated PDP2 and PDP2m from U2OS cell lysates. Tubulin controls in (a) and (b) are taken from the supernatant (superntnt.). c) In vitro disruption of the indicated PP1 holoenzymes by 20 μm of peptides, releasing the PP1 catalytic subunit from the PIP immunoprecipitates. Control: TBS buffer. The results are presented as the mean±standard deviation (*n*=3).

To understand in detail how **PDP2** interacts with PP1, we solved the crystal structure of the PP1:**PDP2** complex (Figure 4) at a resoluion of 3.1 Å. The structure shows that **PDP2** binds in the same groove of PP1 (Figure 4 a) as other structurally characterized PIPs containing the RVxF motif.^[8,^^13]^ Thirteen residues at the C-terminus of **PDP2** (1-NARVTFAEAAEII-23) are observed in an extended conformation, with Arg13, Val14, Thr15, Ala17, Glu21, and Ile23 hydrogen bonding with backbone atoms of PP1. As in previous structures,^[8,^^14]^ Val14 and Phe16 are buried in the binding groove and Arg13 interacts with PP1 Asp242 (Figure 4 b). The C-terminal isoleucine makes van der Waals contacts with PP1 Leu296, suggesting why alanine mutation of this residue resulted in a decreased potency. Hydrophobic contacts with PP1 Leu296 are also observed in the PP1:spinophilin complex.^[14]^ Mutation of the basic N-terminal residues of **PDP2** led to a decreased potency, but these residues are not observed in the crystal structure. However, the position of the visible N-terminal residues of **PDP2** suggests that these basic residues are located adjacent to a negatively charged region on the surface of PP1 (Figure 4 a). Thus, electrostatic interactions between this acidic region of PP1 and the basic N-terminus of **PDP2** may additionally stabilize the complex.

**Figure 4:**
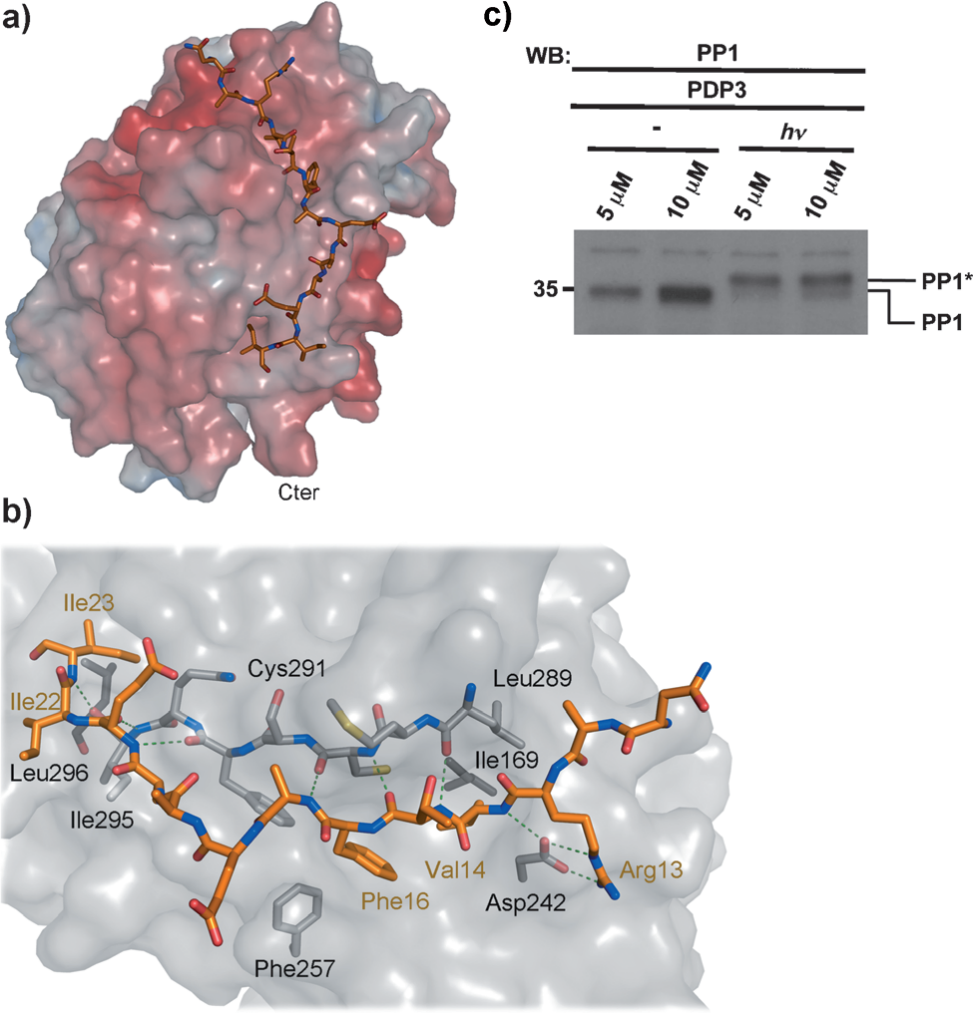
Demonstration of the direct interaction of PDP2 and PDP3 with PP1. a) Crystal structure of PDP2 bound to the acidic molecular surface of PP1. The surface is colored according to electrostatic potentials calculated by APBS.^[15]^ The potential scale ranges from −10 kT e^−1^ (red) to 10 kT e^−1^ (blue).^[15]^ The coordinates are deposited in the protein data bank (PDB) with the access code 4G9J. b) Detailed view of interactions between PDP2 (yellow) and PP1 (gray). Hydrogen bonds are shown in green. The stereoimage of the electron density around the peptide is shown in Figure S10 in the Supporting Information. c) Immunoblot of PP1 in a streptavidin pull-down of biotinylated PDP3 from U2OS cell lysate with and without irradiation with UV light (365 nm) (see the Supporting Information). PP1* denotes PP1 cross-linked to PDP3. The upper band is a nonspecific band.

To validate the physical association of **PDP2** with PP1 in cell lysates, we designed photo-cross-linkable analogues by substituting Phe17 and Ala18 of **PDP2** with L-4-benzoylphenylalanine (*Bpa*).^[16]^ The analogues were biotinylated to enable pull-down of the cross-linked complex out of the cell lysates. *Bpa* substitution at Phe17 failed to yield any cross-linked complex (Figure S11 in the Supporting Information). The peptide resulting from *Bpa* replacement of Ala18, **PDP3** (Table 1), covalently cross-linked to PP1 upon irradiation with ultraviolet light (365 nm) in a dose-dependent manner (Figure 4 c), proving the direct physical interaction of PP1 with **PDP3** in cell lysates. The efficient cross-linking of **PDP3** to PP1 can be explained from the PP1:**PDP2** crystal structure, where Met290 of PP1 is in close proximity to Ala18 which, when replaced by *Bpa*, presumably results in the formation of a covalent bond between *Bpa* and the Met290 side chain. As **PDP3** covalently captured PP1 from cell lysates, we determined if it could also be employed in intact cells. Different cells took up FAM-**PDP3** and FAM-**PDP3m** equally well (Figure S12), and FAM-**PDP3** showed penetration properties similar to those of FAM-**PDP2** (Figure S13). Like **PDP2**, biotinylated **PDP3** did not co-precipitate PP2A (Figure S14).

Next, we explored the effects of **PDP**s on PP1 in intact cells. To this end, we first tested whether treating cells with **PDP**s would have an effect on the mitotic phosphorylation of histone H3 on threonine 3 (T3), a well-established PP1 substrate^[17]^ (Figure 5 a). Surprisingly, addition of **PDP2** did not show any effect, even when a fivefold excess of **PDP2** was employed relative to **PDP3** (Figure S15 in the Supporting Information). On the contrary, **PDP3** treatment promoted histone H3T3 dephosphorylation even though **PDP3** has lower in vitro potency than **PDP2**, and a clear dose–response relationship was observed (Figure 5 b). We also tested whether biotinylation would have an effect. For acetylated and biotinylated peptides, similar behavior was observed (Figure 5 a). Since **PDP2** and **PDP3** penetrated cells to a similar extent, the better efficiency of **PDP3** cannot be attributed to its better cell-penetration properties. Therefore, the higher efficiency of **PDP3** could be explained by either a higher intracellular stability or its intrinsic cross-linking properties owing to the presence of *Bpa*, or a combination of both. However, the intrinsic cross-linking property of **PDP3** can be ruled out based on our observation that incubation of **PDP3** with cell lysates did not yield any cross-linked PP1 in the absence of UV irradiation (Figure 4 c).

**Figure 5:**
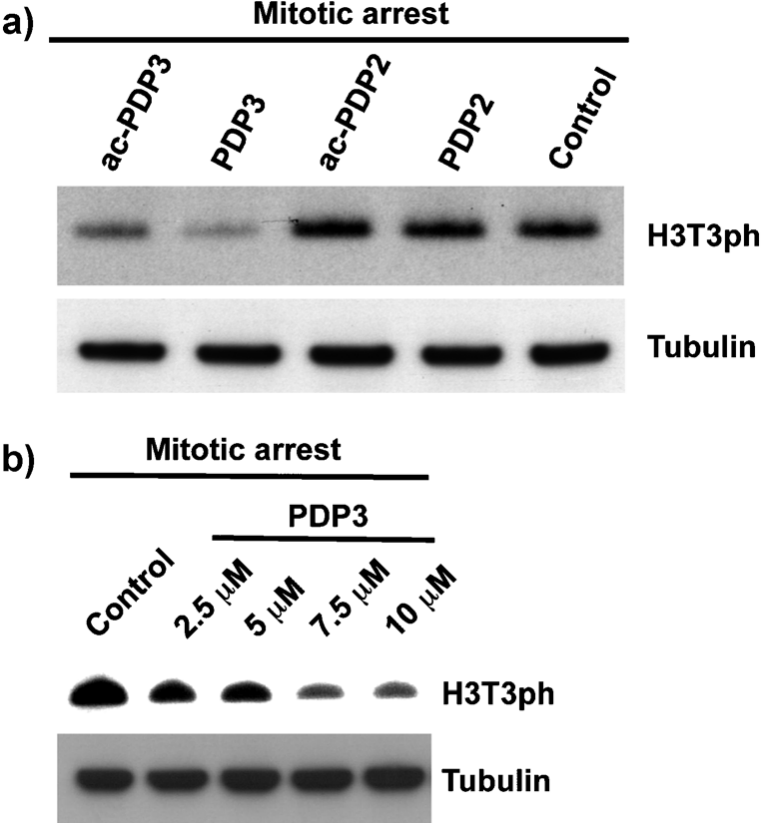
In-cell activity of peptides on histone H3T3 phosphorylation mediated by PP1. a) Immunoblot of total cell lysates prepared after treatment of intact U2OS cells with 10 μm of acetylated (ac-) and biotinylated peptides (no prefix) and a buffer control for 3 h during mitotic arrest (see the Supporting Information). b) Immunoblot of the concentration dependence of PDP3 activity under the same conditions as in (a).

To examine the hypothesis of increased stability, we compared the in-cell stability of FAM-**PDP2** and FAM-**PDP3**. Intact U2OS cells (in mitotic arrest, to apply the same conditions as before) were incubated with various concentrations of FAM-**PDP2** and FAM-**PDP3** for 3 h and lysed to determine the remaining amount of the intact peptides by fluorescence in-gel scan (see the Supporting Information). The results clearly demonstrated that FAM-**PDP3** was significantly more stable than FAM-**PDP2** inside cells (Figure 6). This corroborated our hypothesis that the substitution of the canonical amino acid (Ala) in **PDP2** with an unnatural amino acid (*Bpa*) greatly enhanced the cellular stability of **PDP3**. Since **PDP3** is more stable than **PDP2** inside cells, whereas **PDP2** is more potent in vitro, and since their cell-penetration properties are similar, this finding also proves that **PDP3** exerts its effect after penetrating the cells and not by adhering to the cell surface. Furthermore, our results demonstrate the difficulty in transferring probes, in particular peptidic ones, from an in vitro to an in-cell setting, as unexpected effects can occur.

**Figure 6:**
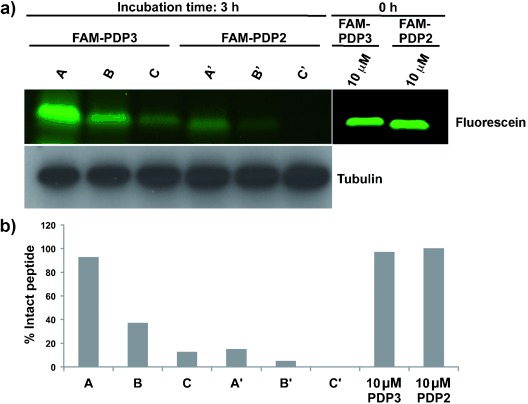
Cellular stability of FAM-PDP2 and FAM-PDP3. a) Fluorescence readout of total cell lysates run on a gel after incubation of U2OS cells during mitotic arrest with the peptides for 3 h, subsequent washing, and cell lysis (see the Supporting Information). Initial peptide concentrations: A) 10 μm, B) 5 μm, and C) 1 μm of FAM-PDP3; A′) 10 μm, B′) 5 μm, and C′) 1 μm of FAM-PDP2. b) Fluorescent bands were quantified after normalization with the tubulin control. Shown is a representative experiment out of two.

Based on this finding, we used **PDP3** for further experiments in living cells. As there were no differences in potency observed between biotinylated and acetylated forms (Figure 5 a), the biotinylated peptide was selected, enabling detection of the peptide in cells by immunofluorescence to further confirm and ensure **PDP3**s cell penetration ability (Figure S16 in the Supporting Information). First, we investigated disruption of PP1 holoenzymes by **PDP3** in living cells. Following incubation of U2OS cells for 3 h with 10 μm **PDP3**, cell lysis, and analysis (see the Supporting Information), three of the five endogenous PIPs examined showed a reduced association with PP1 (Figure 7 a). These results demonstrate the efficacy of **PDP3** in disrupting PP1 holoenzymes inside cells.

**Figure 7:**
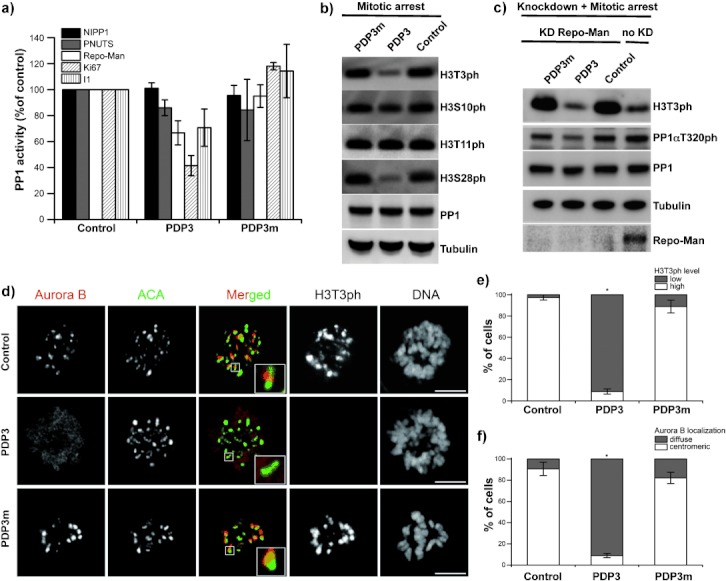
PDP3 efficacy and effects in living cells. a) Disruption of PP1 holoenzymes after incubation of U2OS cells arrested in mitosis with 10 μm of peptides for 3 h and subsequent cell lysis. Control: TBS buffer. The results are presented as the mean±standard deviation (*n*=3). b) Immunoblots of U2OS cells treated as in (a). c) Immunoblot of cells as in (b) after Repo-Man knockdown (KD). d) Confocal images of U2OS cells in mitotic arrest after incubation for 1 h with vehicle control (dimethyl sulfoxide), 40 μm PDP3, and 40 μm PDP3m before fixation. Higher concentrations of PDP3 and a shorter time point than in previous experiments were used to induce an acute effect that is clearly visible under the immunofluorescence conditions. ACA is a kinetochore marker. Scale bars represent 5 μm. e) Quantification of the changes in H3T3ph levels and f) quantification of Aurora B localization after peptide treatment. The results are represented as the mean±standard deviation (*n*=3); *P<0.01, Student’s t test.

Next, we examined the effects of **PDP3** on the mitotic phosphorylation of histone H3 on additional sites that are known to be dephosphorylated by PP1.^[17]^ **PDP3** decreased the phosphorylation of histone H3 on Thr3 and Ser28, but did not significantly affect the phosphorylation of Ser10 and Thr11 (Figure 7 b). This is in accord with published data showing that the dephosphorylation of H3T3 and H3S28 in vitro proceeds with a lower concentration of PP1 than that required for the dephosphorylation of H3S10 and H3T11.^[17]^ **PDP3m** did not affect histone H3 phosphorylation (Figure 7 b), suggesting that **PDP3** accelerates the dephosphorylation of selected residues on histone H3 in living cells by binding to PP1.

Despite having a cell-penetrating stretch, neither **PDP3** nor **PDP3m** showed any cytotoxicity under the experimental conditions. However, when applying prolonged incubation times we observed cytotoxic effects of **PDP3** but not **PDP3m**, showing that these effects can be attributed to increased PP1 activity inside cells and, notably, are not due to nonspecific toxicity of the polybasic stretch (Figure S17 in the Supporting Information).

During mitosis, H3T3ph is dephosphorylated by a complex of PP1 and the histone H3 PP1-targeting subunit Repo-Man.^[17]^ Since **PDP3** also disrupted the PP1:Repo-Man complex (Figure 7 a), our data suggest that histone H3 can be dephosphorylated by nontargeted, **PDP3**-associated PP1. This suggestion was corroborated by examining the effects of **PDP3** on mitotic H3T3ph after the siRNA-mediated knockdown of Repo-Man (Figure 7 c). A deficiency of Repo-Man resulted in a hyperphosphorylation of H3T3, but this was reversed by preincubation with **PDP3**, without any effect on the total level of PP1. **PDP3** also induced the dephosphorylation of PP1 at Thr320 (Figure 7 c). This site is reported to be auto-dephosphorylated by PP1, resulting in partial activation of PP1 in a cell-cycle-dependent manner.^[18]^ Thus, in addition to disrupting PP1 inhibitory holoenzymes, Thr320 dephosphorylation can be another mechanism by which **PDP3** activates PP1.

We next studied the downstream effects of enhanced histone H3 dephosphorylation by **PDP3** during mitosis. H3T3ph serves as a docking site for Survivin, a regulatory subunit of Aurora B kinase, and mediates the targeting of this essential mitotic kinase to the centromeres during prometaphase.^[19]^ Consistent with this mitotic function of H3T3 phosphorylation, we found using immunofluorescence that **PDP3**, but not **PDP3m**, caused the centromeric loss of both H3T3ph and Aurora B in mitotically arrested U2OS cells (Figure 7 d–f). Similar effects have also been described after the knockdown of the H3T3 kinase Haspin^[19]^ and the overexpression of Repo-Man.^[17]^ Collectively these results demonstrate that **PDP3** activates PP1 in living cells.

In conclusion, we have developed a cell-permeable peptide (**PDP3**) that competes with endogenous RVxF-containing PIPs for binding to PP1 in living cells, but does not bind the closely related phosphatase PP2A. Our design strategy demonstrates that a simple modification of peptides with an unnatural amino acid can impart remarkable in-cell stability to peptides. In addition, we show that carefully designed cell-penetrating peptides do not show nonspecific cytotoxicity as a result of the polybasic stretch. Our results demonstrate that, together with cell-penetrating properties, the balance between in vitro activity (**PDP3** has higher EC_50_ than the precursor peptides) and in-cell stability is crucial for peptidic probes. We observed that by blocking PIP–PP1 interactions and promoting auto-dephosphorylation, **PDP3** generates active PP1 that efficiently dephosphorylates a subset of PP1 substrates. In accordance with biochemical and structural studies, our data reveal that a major function of PIPs is to restrain PP1 from dephosphorylating proteins in an uncontrolled manner.^[7,^^20]^ Our study also shows that it is possible to selectively activate PP1 in living cells, opening up new routes to decipher PP1 signaling.^[3]^ The fact that addition of **PDP3** leads to the same outcome on H3T3 phosphorylation as treatment with an inhibitor of the H3T3 kinase Haspin^[21]^ suggests that **PDP3** (derivatives) or equivalent small molecules may have therapeutic potential as antagonists of pathologically increased kinase signaling. In addition, these compounds potentially can be used as sensitizers of kinase inhibitors that are used clinically. Furthermore, the crystal structure of PP1:**PDP2** can serve as a template for the design of further potent peptide/peptidomimetic analogues to disrupt PP1–PIP interactions.

## Supporting Information

Detailed facts of importance to specialist readers are published as ”Supporting Information”. Such documents are peer-reviewed, but not copy-edited or typeset. They are made available as submitted by the authors.

## References

[1] McConnell JL, Wadzinski BE (2009). Mol. Pharmacol.

[2] McCluskey A, Sim ATR, Sakoff JA (2002). J. Med. Chem.

[3] Zorn JA, Wells JA (2010). Nat. Chem. Biol.

[4] Tappan E, Chamberlin AR (2008). Chem. Biol.

[5] Ceulemans H, Bollen M (2004). Physiol. Rev.

[6] Bollen M, Peti W, Ragusa MJ, Beullens M (2010). Trends Biochem. Sci.

[7] Hendrickx A, Beullens M, Ceulemans H, Den Abt T, Van Eynde A, Nicolaescu E, Lesage B, Bollen M (2009). Chem. Biol.

[8] Egloff MP, Johnson DF, Moorhead G, Cohen PT, Cohen P, Barford D (1997). EMBO J.

[9] Beullens M, Van Eynde A, Vulsteke V, Connor J, Shenolikar S, Stalmans W, Bollen M (1999). J. Biol. Chem.

[10] Walensky LD, Kung AL, Escher I, Malia TJ, Barbuto S, Wright RD, Wagner G, Verdine GL, Korsmeyer SJ (2004). Science.

[12] Ter-Avetisyan G, Tünnemann G, Nowak D, Nitschke M, Herrmann A, Drab M, Cardoso MC (2009). J. Biol. Chem.

[13] Wakula P, Beullens M, Ceulemans H, Stalmans W, Bollen M (2003). J. Biol. Chem.

[14] Ragusa MJ, Dancheck B, Critton DA, Nairn AC, Page R, Peti W (2010). Nat. Struct. Mol. Biol.

[15] Baker NA, Sept D, Joseph S, Holst MJ, McCammon JA (2001). Proc. Natl. Acad. Sci. USA.

[16] Stewart ML, Fire E, Keating AE, Walensky LD (2010). Nat. Chem. Biol.

[17] Qian J, Lesage B, Beullens M, Van Eynde A, Bollen M (2011). Curr. Biol.

[18] Wu JQ, Guo JY, Tang W, Yang CS, Freel CD, Chen C, Nairn AC, Kornbluth S (2009). Nat. Cell Biol.

[19] Kelly AE, Ghenoiu C, Xue JZ, Zierhut C, Kimura H, Funabiki H (2010). Science.

[20] Ragusa MJ, Allaire M, Nairn AC, Page R, Peti W (2011). FEBS Lett.

[21] Huertas D (2012). Oncogene.

