# Supplementary material for: Development of a Peptide that Selectively Activates Protein Phosphatase-1 in Living Cells
Source: Angew Chem Int Ed Engl. 2012 Sep 7;51(40):10054–9. doi: 10.1002/anie.201204308 (PMC3531619; doi:10.1002/anie.201204308)
Supplement: Supplementary file 1 [file anie0051-10054-SD1.pdf]

Angewandte Chemie
Eine Zeitschrift der Gesellschaft Deutscher Chemiker

Supporting Information

© Wiley-VCH 2012

69451 Weinheim, Germany

Development of a Peptide that Selectively Activates Protein Phosphatase-1 in Living Cells**

Jayanta Chatterjee, Monique Beullens, Rasa Sukackaite, Junbin Qian, Bart Lesage, Darren J. Hart, Mathieu Bollen, and Maja Köhn**

anie_201204308_sm_miscellaneous_information.pdf

Table of contents

Supporting results 3

Supporting methods 15

Supporting references 21

Supporting results

Peptide design

Supporting Table 1. Peptides described in the manuscript. All peptides (except 5-carboxyfluorescein- and biotin- labeled) are acetylated at the *N* terminus. All peptides carry an amide group at the *C* terminus. MW: molecular weight obtained from MALDI. *Bpa*: L-Benzoylphenylalanine.

Peptide	*Sequence*	*MW*
**PDP0**	RPKRKRKNSRVTFSEDDEII	2515.8
**PDP1**	RPKRKRKNARVTFAEAAEII	2395.9
**PDP2**	RRKRPKRKRKNARVTFAEAAEII	2836.6
**PDP2m**	RRKRPKRKRKNARATAAEAAEII	2734.8
**PDP3**	RRKRPKRKRKNARVTF*Bpa*EAAEII	3018.9
**PDP3m**	RRKRPKRKRKNARATA*Bpa*EAAEII	2914.7

Supporting Table 2. Peptides described in the Supporting Section. MW: molecular weight obtained from MALDI. *Dap*: L-2,3-Diaminopropionic acid; p: D-Pro.

Peptide	*Sequence*	*MW*
**PDP4**	**A**PKRKRKNSRVTFSEDDEII	2430.7
**PDP5**	R**A**KRKRKNSRVTFSEDDEII	2489.8
**PDP6**	RP**A**RKRKNSRVTFSEDDEII	2458.7
**PDP7**	RPK**A**KRKNSRVTFSEDDEII	2430.7
**PDP8**	RPKR**A**RKNSRVTFSEDDEII	2458.7
**PDP9**	RPKRK**A**KNSRVTFSEDDEII	2430.7
**PDP10**	RPKRKR**A**NSRVTFSEDDEII	2458.7
**PDP11**	RPKRKRK**A**SRVTFSEDDEII	2472.8
**PDP12**	RPKRKRKN**A**RVTFSEDDEII	2499.8
**PDP13**	RPKRKRKNS**A**VTFSEDDEII	2430.7
**PDP14**	RPKRKRKNSR**A**TFSEDDEII	2487.7
**PDP15**	RPKRKRKNSRV**A**FSEDDEII	2485.8
**PDP16**	RPKRKRKNSRVT**A**SEDDEII	2437.7
**PDP17**	RPKRKRKNSRVTF**A**EDDEII	2499.8
**PDP18**	RPKRKRKNSRVTFS**A**DDEII	2457.7
**PDP19**	RPKRKRKNSRVTFSE**A**DEII	2471.8
**PDP20**	RPKRKRKNSRVTFSED**A**EII	2471.8
**PDP21**	RPKRKRKNSRVTFSEDD**A**II	2457.7
**PDP22**	RPKRKRKNSRVTFSEDDE**A**I	2473.7
**PDP23**	RPKRKRKNSRVTFSEDDEI**A**	2473.7
**PDP24**	RPKRKRKN**A**RVTF**A**EDDEII	2482.5
**PDP25**	RPKRKRKNSRVTFSE**AA**EII	2427.6

**PDP26**	RPKRKRKNSRVTFS**A**DD**A**II	2399.8
**PDP27**	RPKRKRKN**A**RVTF**A**E**A**DEII	2438.3
**PDP28**	RPKRKRKNSRVTFS**A**D**AA**II	2355.5
**PDP29**	RPKRKR**pP**SRVTFSEDDEII	2466.2
**PDP30**	RPKRKRK**pP**RVTFSEDDEII	2507.1
**PDP31**	RPKRKR**pPA**RVTF**A**E**A**DEII	2391.5
**PDP32**	RPKRKR**pPA**RVTF**A**E**AA**EII	2346.5
**PDP33**	*cyclo*(CAAVAAp*Dap*ARVTFC)EAAEII	2029.7
**PDP34**	*cyclo*(CAAVAAp*Dap*(KRKRKPR)ARVTFC)EAAEII	3022.8
**b-PDP35**	Biotin-RPKRKRKN**A**RVT***Bpa*A**E**AA**EII	2683.3
**b-PDP36**	Biotin-RPKRKRKN**A**RVTF***Bpa***E**AA**EII	2758.9

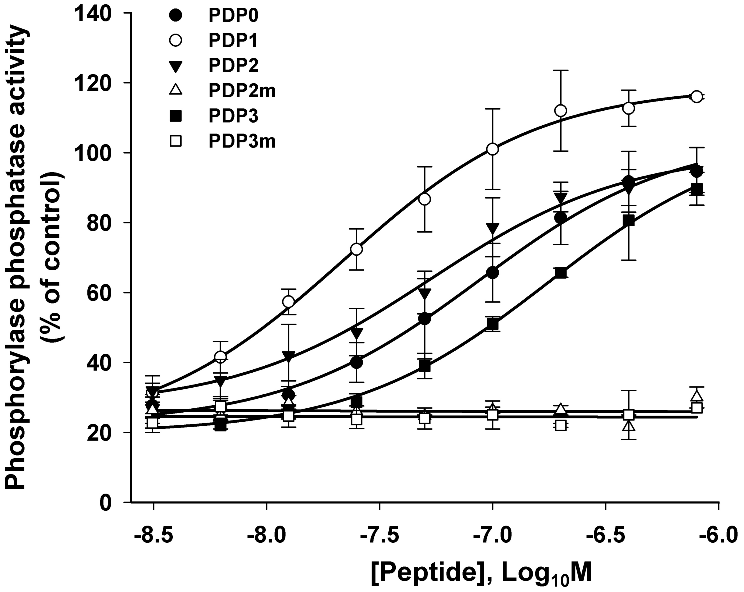

Supporting Figure 1. Deinhibition of the PP1:Inhibitor2 complex by increasing concentrations of the indicated peptides. This Figure shows the data corresponding to Table 1 in the manuscript.

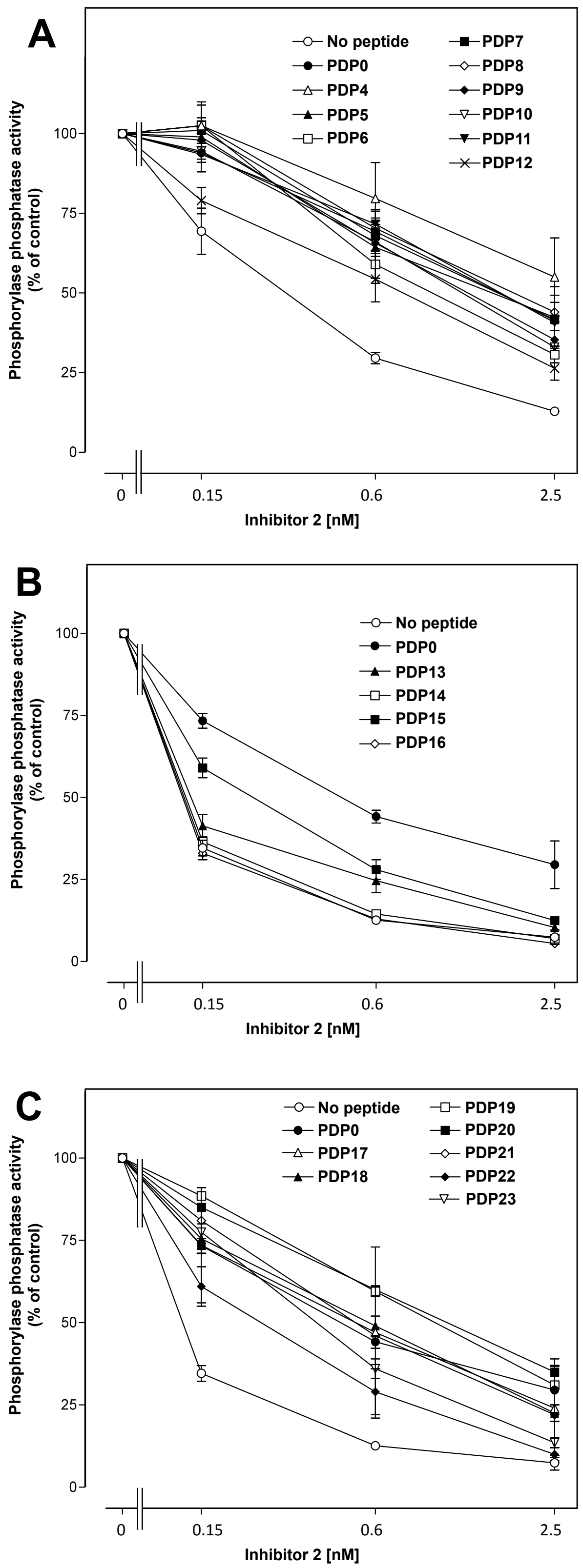

Supporting Figure 2. Deinhibition of PP1 against increasing concentrations of I2 (Inhibitor 2) with 50 nM of Ala-mutants of NIPP1(191-210) (**PDP0**) in the Ala-scan. A) *N*-terminal to the RVTF-motif, B) RVTF-motif, and C) *C*-terminal to the RVTF-motif. The results are presented as means ±S.E.M. (n= 3-4).

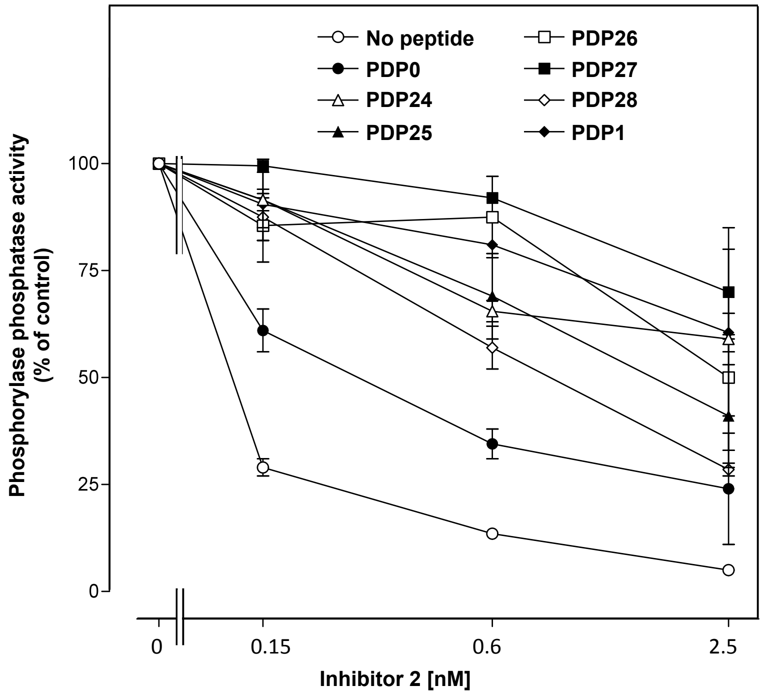

Supporting Figure 3. Deinhibition of PP1 against increasing concentrations of I2 with 50 nM of alanine-mutants of **PDP0**. The deinhibition assays show that alanine replacement of Ser199, Ser204 and Asp206 (**PDP27**) or Ser199, Ser204, Asp206, and Asp207 (**PDP1**) have the biggest positive impact on the efficacy of the peptide. The results are presented as means ±S.E.M. (n= 4).

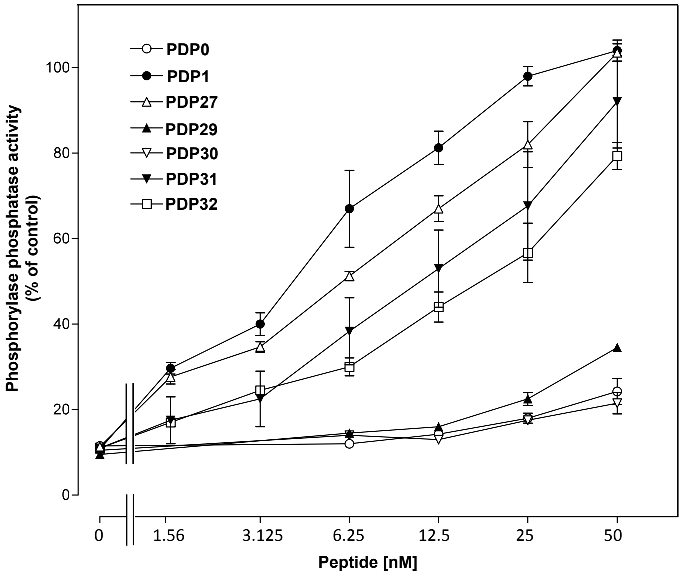

Supporting Figure 4. Dose-dependent deinhibition of the PP1:I2 complex by alanine-mutated and D-Pro–L-Pro analogues of **PDP0**. To improve the affinity of **PDP0**, we designed β-hairpin peptides (**PDP29** and **PDP30**), which are D-Pro–L-Pro templated analogues of **PDP0,** as RVTF-motifs of the regulatory subunits of PP1 bind in an extended conformation to PP1. There was a slight increase in the efficacy of **PDP29** in comparison to **PDP0** but with

PDP30 there was no significant change. However, when Ser199, Ser204 and Asp206 (**PDP31**) or Ser199, Ser204, Asp206 and Asp207 (**PDP32**) were replaced by alanine, in addition to the D-Pro–L-Pro motif, there was a substantial enhancement in the efficacy of these two peptides. Notably, the flexible linear peptides **PDP27** and **PDP1** were still the strongest competitors of I2. The results are presented as means ±S.E.M. (n= 3).

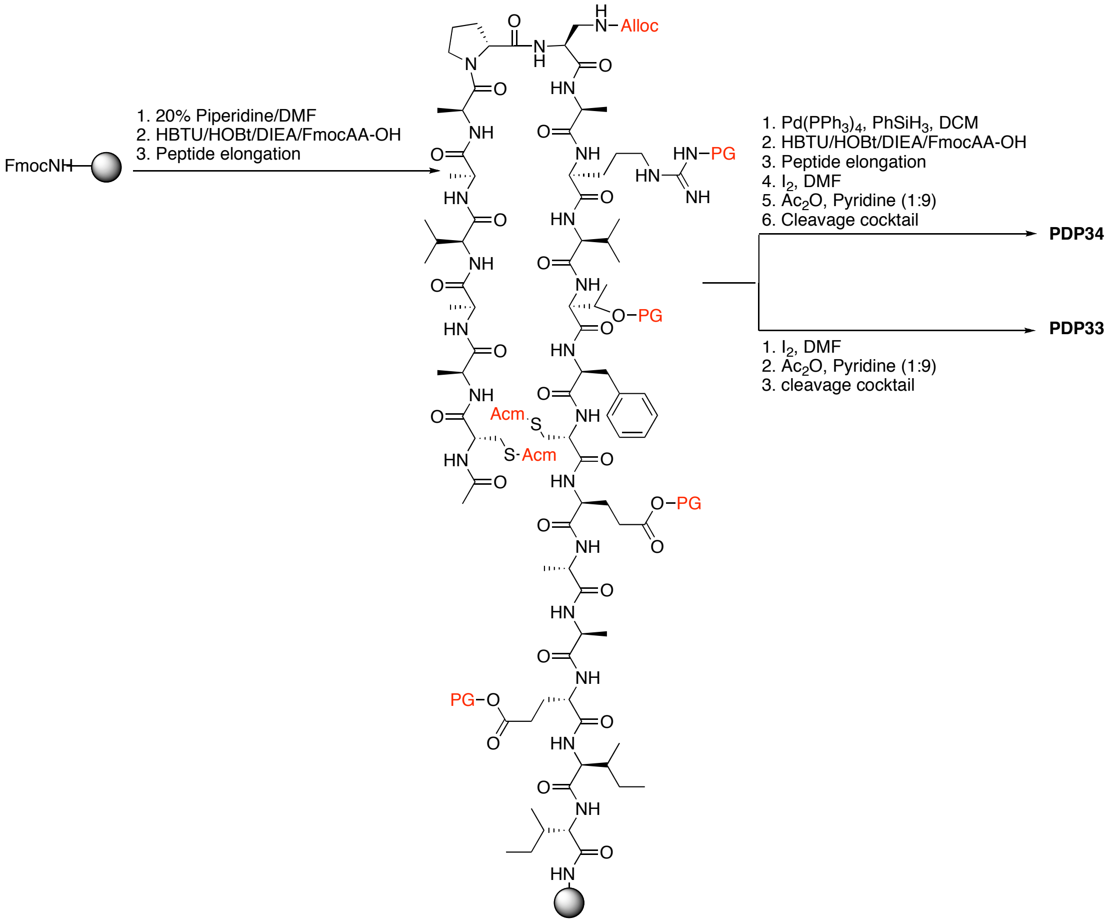

Supporting Scheme 1. Schematic representation for the synthesis of **PDP33** and **PDP34** on solid support. PG refers to standard protecting groups. In addition to the linear β-hairpin peptides, we also designed and synthesized disulfide bridged cyclic peptides (**PDP33** and **PDP34**), to preorient the RVTF-motif in a stabilized extended conformation and to study the impact of the stabilized conformation of RVTF-motif on binding to PP1.

a

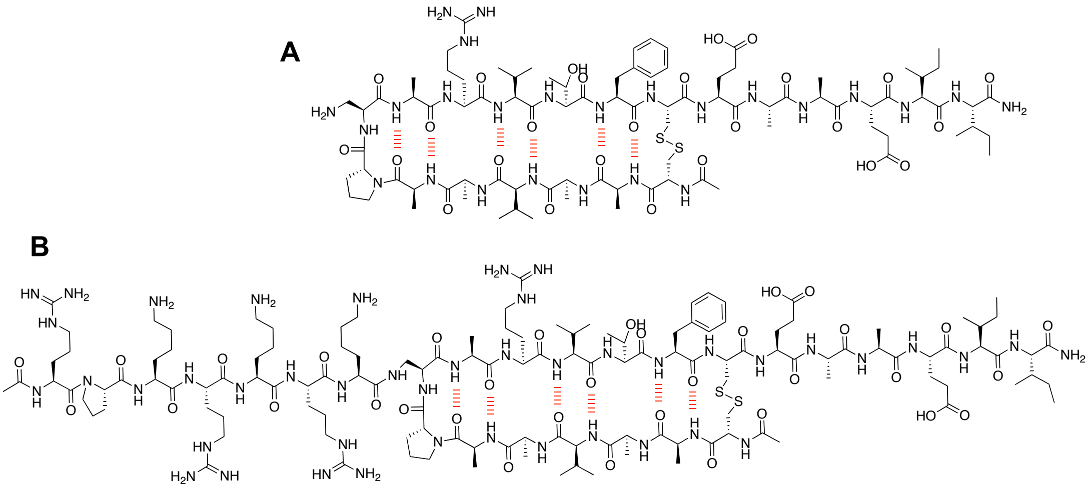

b

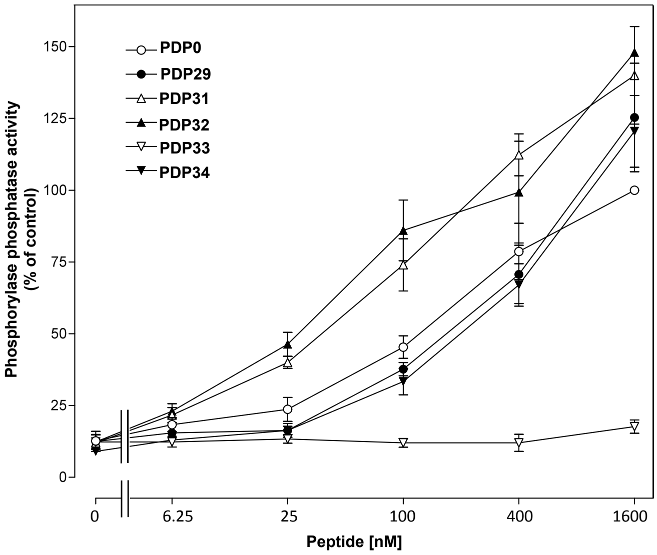

Supporting Figure 5. a) Disulfide-bridged cyclic peptides **A**) **PDP33** and **B**) **PDP34**. **PDP33** is a modified derivative of **PDP32**, where the amino acids *N*-terminal to D-Pro-L-*Dap* (Diaminopropionic acid) have been replaced by L-Ala and L-Val to stabilize the conformation and for ease of synthesis. We replaced L-Pro with L-*Dap* to have a further possibility of functionalization of the side chain. **PDP34** is a branched cyclic analogue wherein the L-*Dap* side chain of **PDP33** was functionalized to mimic the polybasic sequence of **PDP0**. b) Dose-dependent deinhibition of the PP1-I2 complex by increasing concentrations of D-Pro–L-Pro and disulfide-bridged cyclic peptides. In spite of the presence of the RVTF-motif in a presumably extended conformation, **PDP33** did not show any improvement in the efficacy. It should be noted that there is considerable sequence similarity between **PDP34** and **PDP1**; however, there was a remarkable difference between the efficacies of these two peptides, with a very low affinity of **PDP34** towards PP1. The results are presented as means ±S.E.M. (n= 3).

Cellular uptake

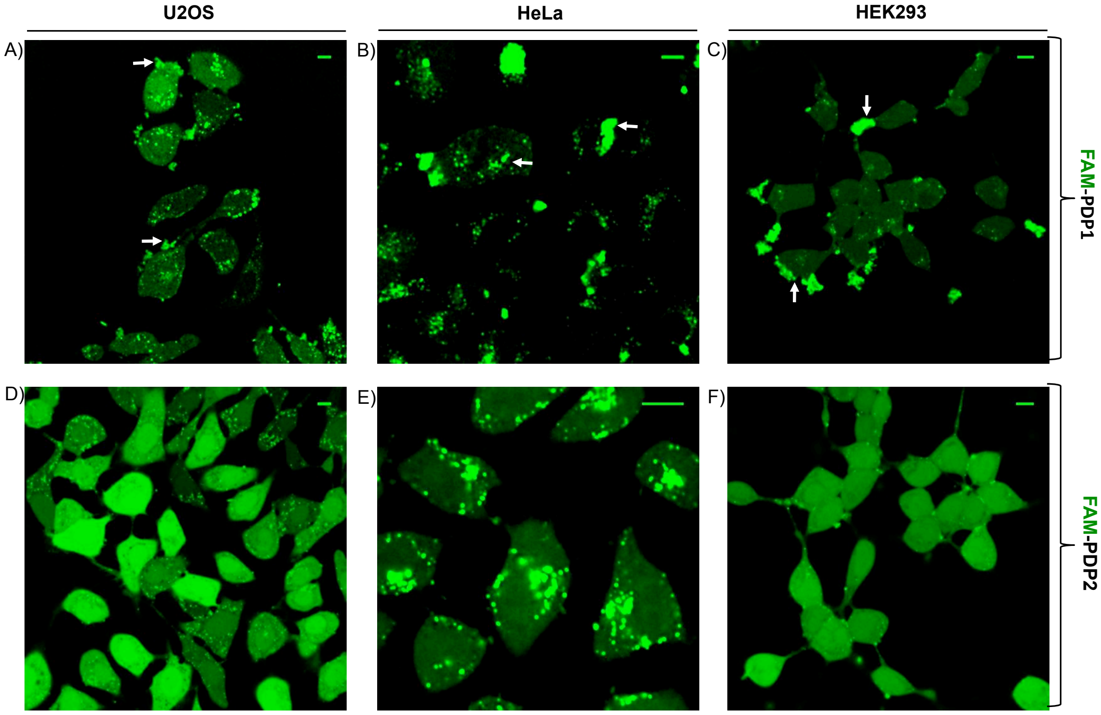

Supporting Figure 6. Confocal microscope images of 5-Carboxyfluorescein (FAM)-labeled **PDP1** and **PDP2**. 100 µM FAM-**PDP1** was added to A) U2OS cells, B) HeLa cells, and C) HEK293 cells, and incubated at 37 °C for 4 h. Note the aggregation of FAM-**PDP1** in all the cell types, as denoted by arrows. 100 µM FAM-**PDP2** was added to D) U2OS cells, E) HeLa cells, and F) HEK293 cells and incubated at 37 °C for 4 h. The inset scale bars (top right) represent 10 µm.

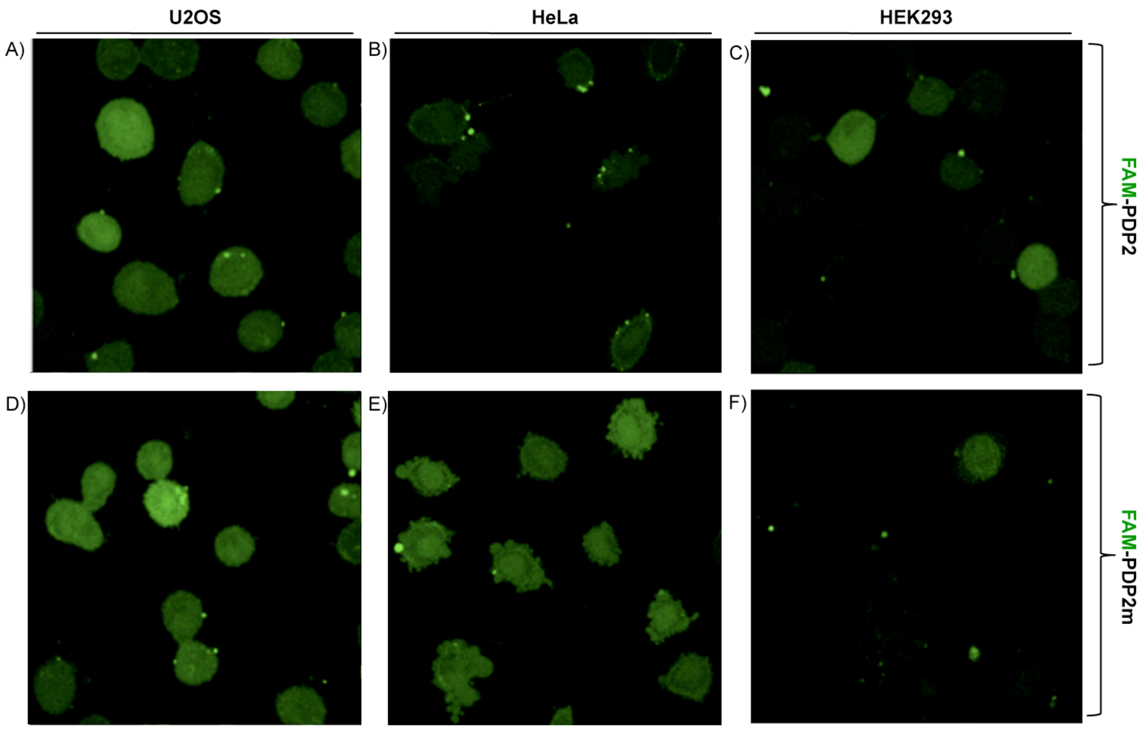

Supporting Figure 7. Confocal microscope images of different cells incubated with 25 μM (compare to Figure 2 in the manuscript) FAM-**PDP2** (A-C) and FAM-**PDP2m** (D-F) at 4 °C for 4 h.

In vitro pull-down of PP1 with biotinylated PDP2 and PDP2m

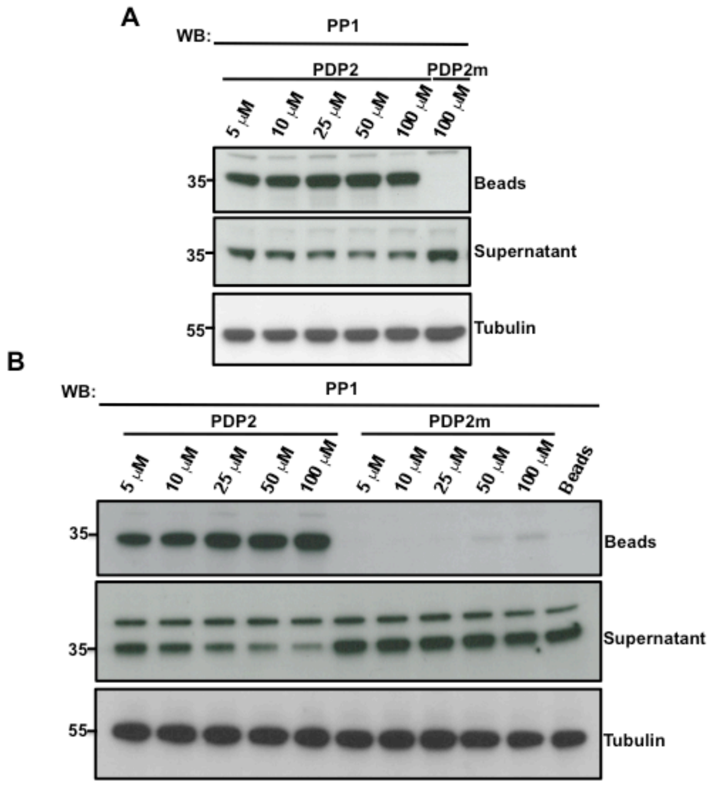

Supporting Figure 8. Dose-dependent in vitro pull-down of endogenous PP1 with

biotinylated peptides. A) From U2OS cell lysate. Note the gradual increase in PP1 associated with the increasing concentration of **PDP2** and concomitant gradual depletion in the supernatant. B) The full immunoblot of **Fig 1c**. The lower band in the middle panel (supernatant panel) is PP1; the upper band is a nonspecific band.

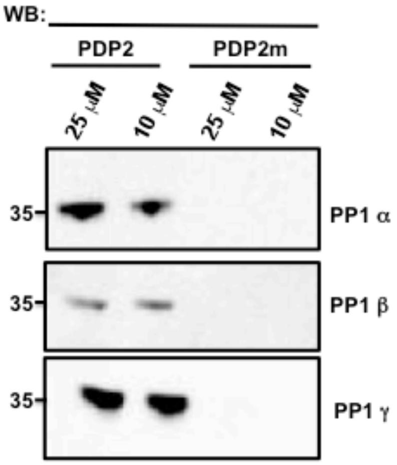

Supporting Figure 9. In vitro pull-down of endogenous PP1 isoforms from cell lysates of U2OS cells with biotinylated peptides. PP1 was visualized by immunoblotting with isoform-specific antibodies. The results show that the ability of **PDP2** to bind PP1 is isoform-independent.

Crystal structure

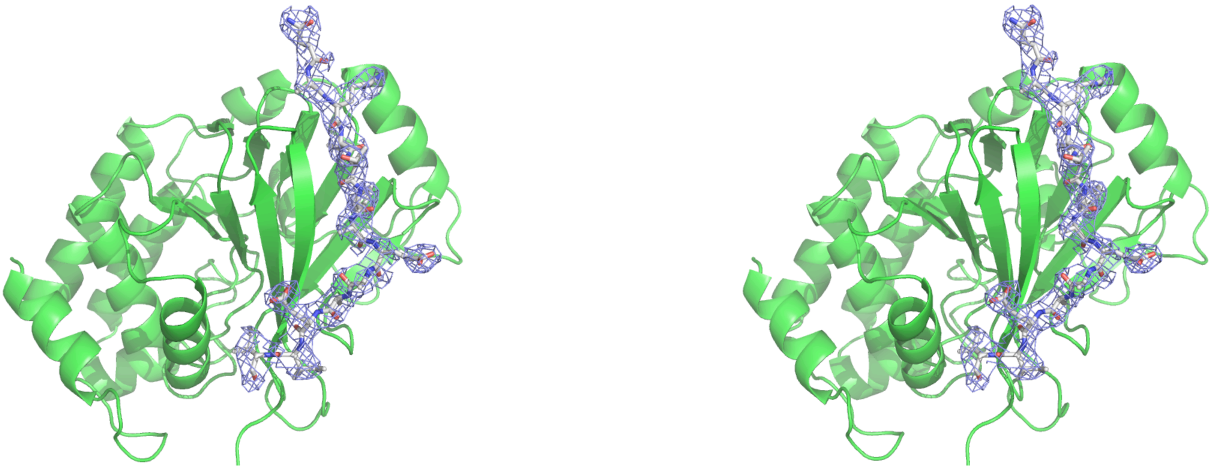

Supporting Figure 10. Stereo-image of the electron density around the peptide **PDP2**.

Development of a photo cross-linkable probe

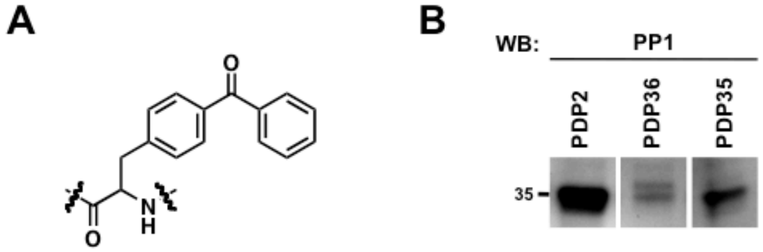

Supporting Figure 11. A) Structure of L-Benzoylphenylalanine (*Bpa*). B) In vitro pull-down of endogenous PP1 and endogenous cross-linked PP1 after incubation and UV irradiation (365 nm) with 10 μM biotinylated-*Bpa* peptides from a U2OS cell lysate. Only **PDP36** shows cross-linking to PP1 (note the band shift), and it displays a lower binding efficiency.

Cellular uptake of the photo cross-linkable probe

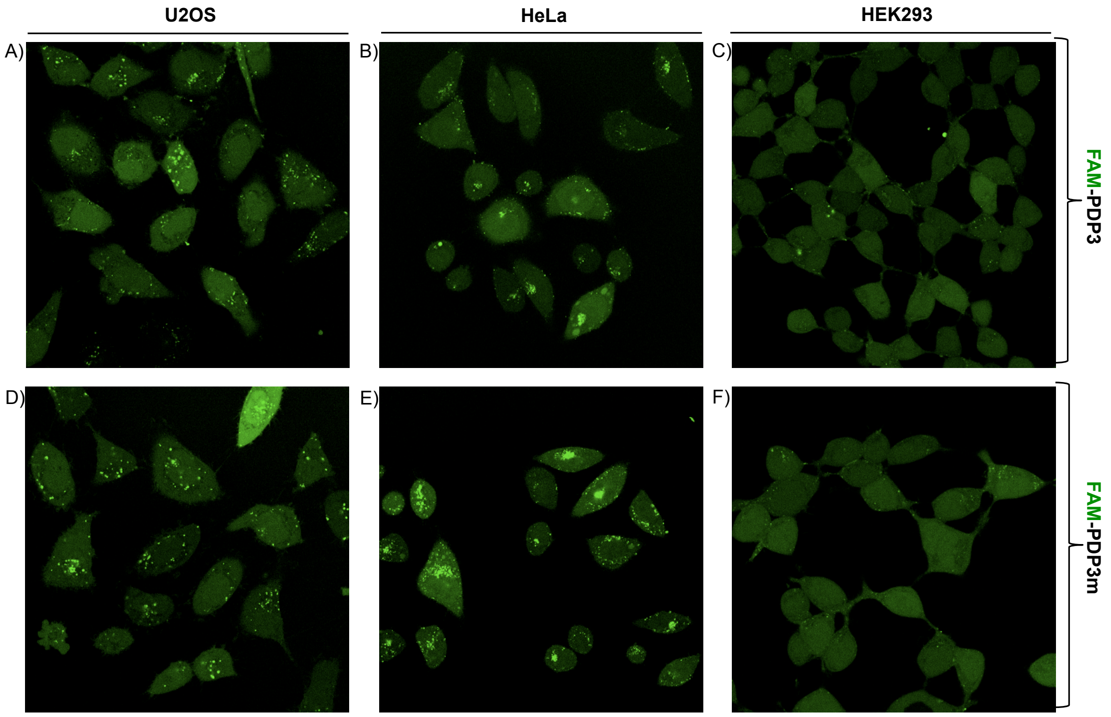

Supporting Figure 12. Confocal microscope images of FAM-labeled **PDP3** and **PDP3m**. 25 µM (compare to Figure 2 in the manuscript) of FAM-**PDP3** was added to A) U2OS cells, B) HeLa cells or C) HEK293 cells, and incubated at 37 °C for 2 h. 25 µM of FAM-**PDP3m** was added to D) U2OS cells, E) HeLa cells or F) HEK293 cells, and incubated at 37 °C for 2 h. Both peptides show similar penetration efficiency.

Comparison between cell-penetrability of FAM-PDP2 and FAM-PDP3

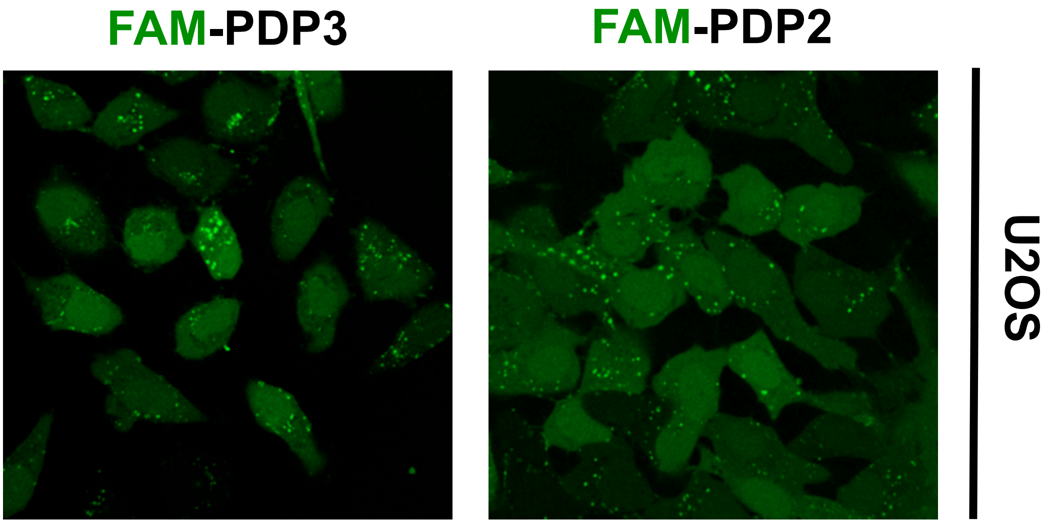

Supporting Figure 13. Confocal microscope images of FAM-**PDP3** and FAM-**PDP2**. 25 μM of each peptides were added to live U2OS cells, incubated at 37 °C for 2 h and imaged live. Both peptides show similar penetration potency.

Selectivity of the photo cross-linkable probe

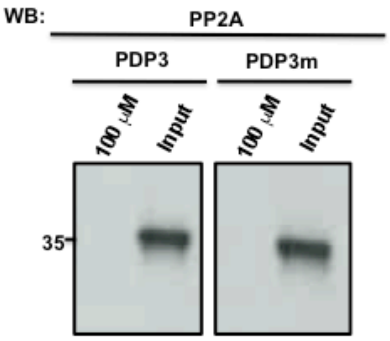

Supporting Figure 14. In vitro pull-down of endogenous PP2A with biotinylated **PDP3** and **PDP3m** from a U2OS cell lysate. No association of PP2A is observed with neither **PDP3** nor **PDP3m**.

Effect of treatment of mitotic cells with the probes

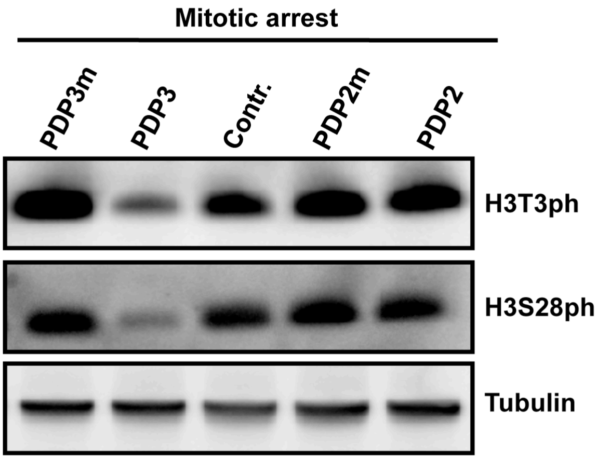

Supporting Figure 15. Immunoblot of total cell lysate prepared after treating U2OS cells with the indicated peptides (50 μM of **PDP2** and **PDP2m**; 10 μM of biotinylated **PDP3** and **PDP3m)** for 3 h during mitotic arrest (first 13 h with nocodazole for mitotic arrest and then additional 3 h with the peptides and nocodazole). Note the decrease in H3T3ph and H3S28ph levels in **PDP3** treated cells.

Cellular localization of biotinylated peptides

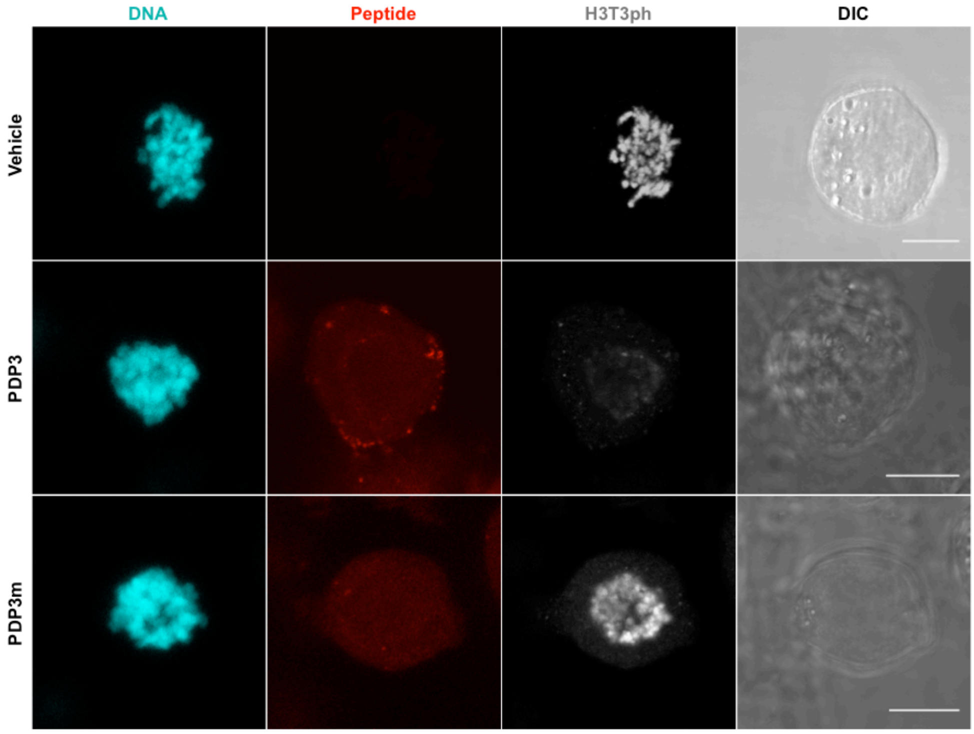

Supporting Figure 16. Confocal images of fixed mitotic arrested U2OS cells treated with 10 μM of biotinylated peptide (as described before). The peptides are quite evenly distributed in cells, although **PDP3** localizes in more punctate structures compared to **PDP3m**. Vehicle: DMSO control. Scale bars represent 10 μm.

Cytotoxicity

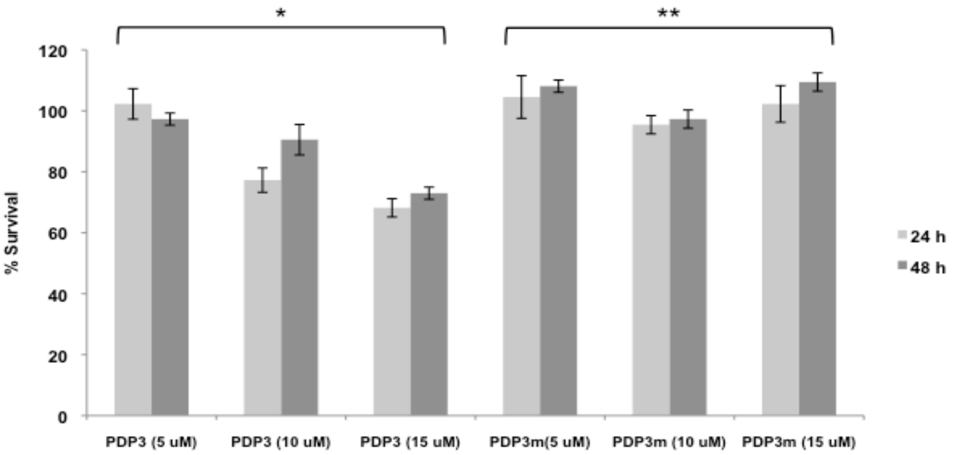

Supporting Figure 17. Proliferation of U2OS cells treated with various concentrations of biotinylated **PDP3** and **PDP3m**. Note the decrease in the cell viability with increasing concentrations of **PDP3** showing the selective cytotoxicity of **PDP3** due to the activation of PP1. Results presented as means ± SD (n=3). *$P < 0.001$ (suggesting statistically significant difference); **$P < 0.2$ (suggesting no statistically significant difference), ANOVA-test.

Supporting methods

Peptide synthesis. Peptides were synthesized manually or on a Multisyntech peptide synthesizer employing a standard Fmoc solid phase peptide synthesis protocol. Double couplings were performed employing Fmoc-amino acids (5 eq), DIEA (N,N-diisopropylethylamine) (6 eq), HBTU (O-benzotriazole-N,N,N',N'-tetramethyl-uronium-hexafluoro-phosphate) (5 eq) and HOBt (N-hydroxybenzotriazole) (5 eq) in 1 ml DMF (N,N-dimethylformamide) using Rink amide resin. The Fmoc deprotection was carried out using 20% piperidine. All peptides (except 5-carboxyfluorescein and biotin labeled) were acetylated at the *N* terminus using 1:9 (vol/vol) acetic anhydride:pyridine. The peptides were cleaved from resin using cleavage cocktail (95% TFA (trifluoroacetic acid): 2.5% TIPS (triisopropylsilane): 2.5% H_2O), ether precipitated, HPLC purified and MS analyzed by MALDI.

Alloc deprotection. The resin was washed and swollen for 15 min with dry DCM and treated with tetrakis(triphenylphosphine)palladium (0.2 eq) and phenylsilane (12 eq) in 1 ml dry DCM (dichloromethane) for 45 min followed by intensive washing with DCM (3x), 0.5% DIEA in DMF (2x), 0.5% sodium diethyldithiocarbamate in DMF (2x) and finally with DCM (3x).

5-Carboxyfluorescein labeling. The *N*-terminally unprotected peptide linked *C*-terminally to the resin was swollen in DMF for 30 min. A solution of 5-carboxyfluorescein (3 eq), HOAt (1-hydroxy-7-azabenzotriazole) (3 eq), HATU ((2-(7-aza-1H-benzotriazole-1-yl)-1,1,3,3-tetramethyluronium hexafluorophosphate) (3 eq), and DIEA (6 eq) in 2 ml DMF was added to the resin and shaken at room temperature for 3 h. The coupling was repeated once to increase the yield of the 5-carboxyfluorescein labeled peptide. Finally, a test cleavage was performed using a cocktail of TFA:TIS:H_2O (95:2.5:2.5) and the completion of the reaction was confirmed by LCMS.

Biotin labeling. The *N*-terminally unprotected peptide linked *C*-terminally to the resin was swollen in NMP (N-methyl-2-pyrrolidone) for 30 min. In a vial biotin (3 eq), HOAt (3 eq), HATU (3 eq) were weighed and dissolved in 2 ml NMP under vigorous shaking. DIEA (6 eq) was added to the solution, and shaken for 3 h. The reaction was repeated once to increase the yield of the biotin-labeled peptide. Finally, a test cleavage was performed using a cocktail of TFA:TIS:H_2O (95:2.5:2.5) and the completion of the reaction was confirmed by LCMS.

Disulfide-bond formation. The peptide-bound resin was transferred to a small reaction vessel and treated with I_2 (10 eq) in DMF (3 ml) for 2 h with vigorous stirring. The resin was thoroughly washed with DMF and DCM, and dried under high vacuum.

Pull-down assays with biotinylated peptide. U2OS, HEK293, or HeLa cells at 90% of confluency (10 cm tissue culture dish) were washed with PBS, trypsinized, harvested with PBS, washed with 1 ml of PBS, and centrifuged at 600 g at 4°C for 10 min. Cell pellets were lysed for 10 min on ice with 400 μl of lysis buffer (50 mM Tris at pH 7.4, 200 mM NaCl, 10 mM EDTA, 20% glycerol, 1% Nonidet P-40, 1 mM phenylmethylsulfonyl fluoride (PMSF), "phosSTOP" (phosphatase inhibitor cocktail tablets from Roche), and "complete mini EDTA free" protease inhibitor cocktail from Roche. Lysates were clarified at 13,000 g for 10 min at 4°C and divided into equal fractions to which biotinylated peptides were added at different concentrations. After rotation with the lysate at 4°C for 2 h, biotinylated peptides were pulled down with streptavidin beads (Streptavidin Sepharose High Performance, GE Healthcare) and washed twice with 750 μl of lysis buffer. Bound proteins and clarified lysates were analyzed by immunoblotting using PP1 (Santa Cruz, sc-7482) and PP2A (Millipore; 05-421) antibodies. The PP1-isoform specific antibodies were purchased from Santa Cruz (PP1α, sc-6104, PP1β, sc-6107, PP1γ, sc-6108).

In vitro cross-linking. After incubation of the clarified lysates with the cross-linkable biotinylated peptide (as described above) in the dark for 2 h at 4°C, the mixture was transferred into a UV transparent, glass bottom 96 well plate (Corning Inc.) and irradiated with 365 nm using a Spectrolinker XL-1500 (Spectronics Corporation) on ice at a distance of

5 cm from the UV lights for different time points. After irradiation, the biotinylated peptides were pulled down with streptavidin beads for additional 2 h and washed stringently six times with 500 μl of lysis buffer to remove any unspecific binding. Finally, the covalently bound PP1 was detected by immunoblotting.

Cellular uptake of peptides. U2OS, HEK293 or HeLa cells were plated at sub-confluency (70-80%) in DMEM supplemented with 10% FCS in a Lab-Tek chamber slide w/cover in an 8-well configuration (Ref 154534, Nalge Nunc International) and incubated at 37°C, 5% CO_2. The following day, the medium was replaced with DMEM (without 10% FCS) containing the indicated concentrations of the fluorescein-labeled peptide and incubated for indicated time points (at 37°C, 5% CO_2 and also on ice at 4°C). Finally, the cells were washed thrice with warm (37°C) PBS and images were obtained at several optical sections (z-stack) at 22°C with a Leica SP2 confocal microscope. Each image is a projection of a 10 μM z-stack collected through a 40X objective. The 488 Argon laser was set at 20% of its total power and the total time for acquiring the entire z-stack was 10 sec. The images were finally processed and assembled using ImageJ 1.45 software (National Institutes of Health). The laser power and image capture settings were constant throughout all the measurements and all the images were subjected to same processing parameters.

Deinhibition phosphatase assay. The activity of rabbit skeletal muscle PP1 [2 nM] was measured with [^{32}P]-labeled glycogen phosphorylase *a* as a substrate in the presence of I2 [1 nM], without or with the indicated concentrations of peptide[1]. In brief, the phosphatase activitiy was derived from the release of radioactively labeled inorganic phosphate (P_i), measured after precipitation of phosphorylase *a* with trichloroacetic acid.

eGFP I3 Construct transfection. Full-length human I3 was introduced between the *Xho*I and *Xma*I sites of pEGFP-C1 (Clontech) yielding an expression vector for EGFP-I3[2].

Immunoprecipitation and disruption of PP1 holoenzyme complexes. Transfection with pEGFP-I3 was carried out with Fugene®-6 Transfection Reagent (Roche Applied Science). Forty-eight hours after transfection, the cells were washed twice with PBS [1.8 mM KH_2PO_4, 8.1 mM Na_2HPO_4, and 150 mM NaCl (pH 7.4)] and harvested in a lysis buffer containing 50 mM Tris-HCl (pH 7.4), 0.3 M NaCl, 0.5% Triton X-100, 0.5 mM phenylmethanesulfonyl fluoride, 1 mM dithiothreitol, 0.5 mM benzamidine, and 5 μM leupeptin. For the immunoprecipitation of endogenous NIPP1 or PNUTS non-transfected U2OS cells were lysed with the same buffer. Following centrifugation (10 min at 6000 g), the supernatants (cell lysates) were used either for the immunoprecipitation with anti-EGFP (Santa Cruz, sc-8334), anti-NIPP1[3], or anti-PNUTS[4] antibodies and protein A-TSK-Sepharose (Affiland). The precipitates were washed once with PBS containing 0.1% NP40 and 0.1 M LiCl and twice

with PBS with 0.1% NP40. For the in vitro disruption assay, the precipitates were incubated with or without the indicated peptide for 30 min at 4°C. After centrifugation (3 min at 6000 g) the protein phosphatase activity released in the supernatant was assayed with glycogen phosphorylase *a* as substrate[1]. For the disruption of PP1 holoenzyme complexes in living cells, U2OS cells were mitotically blocked by nocodazole for 16 h and in the last 3 h of the incubation no peptide or 10 μM of the indicated peptide was added. The cell lysis and the immunoprecipitation were performed as described above. The anti-KI67 antibody (clone MIB-1) was purchased from Dako and the anti-I1 antibody was described previously[5]. After washing, the precipitates were assayed for the presence of PP1 with glycogen phosphorylase *a* as substrate.

Cellular degradation of the peptides. Various dishes containing the same amount of mitotically arrested cells (as described above) were treated with 10 μM, 5 μM, and 1 μM of fluorescently labeled (FAM) **PDP3** and **PDP2**. After 3 h, the cells were washed 3X with warm PBS, scraped, pelleted and total lysates were prepared by boiling with SDS sample buffer. The same volume of the total lysates from different conditions were run on a 12% Bis-Tris gel using MES running buffer along with the inputs for '0' time point = 10 μM each of FAM-**PDP2** and FAM-**PDP3** (final concentration in the same amount volume as used for incubating the mitotic cells) directly loaded on the gel. The fluorescent bands of the peptides corresponding to their appropriate size were visualized using a Fluorescent imager FLA-7000 (Fujifilm). After imaging, the proteins on the SDS-gel were transferred on a nitrocellulose membrane and subjected to western blot against anti-tubulin as loading control. The images were analyzed and quantified using ImageJ 1.45 software (National Institutes of Health).

In-cell PP1 substrate dephosphorylation detected by immunoblots. U2OS cells were cultured consecutively for 24 h with 2 mM thymidine, 2 h without thymidine, and 13 h with 100 nM nocodazole. The cells were carefully washed twice with warm (37 °C) PBS without detaching the mitotically arrested cells, and finally different concentrations of the peptides were added to the cells with 100 nM nocodazole in DMEM, and the cells were incubated additional 3 h under mitotic arrest. The cells were carefully washed 3X with warm PBS, scraped, pelleted and total lysates were prepared by boiling with SDS sample buffer. Equal amounts of total lysates were then run on SDS gel and subjected to Western blot against anti-H3T3ph, H3S10ph, H3T11ph, H3S28ph, PP1aT320ph, PP1, g-Tubulin antibody as described previously[6]. In order to reduce the possibility of mitotic release, which would activate phosphatases and falsify the reults, we avoided any further washing steps.

Repo-Man knockdown and in-cell histone dephosphorylation detected by immunofluorescence. Mitotic arrest, RNAi-mediated knock-down of Repo-Man, in-cell

dephosphorylation of histone H3 and immunostainings were done as described previously[6]. In brief, after siRNA (100 nM) transfection using DharmaFECT (Dharmacon) for 6hr, U2OS cells were cultured consecutively for 24 h with 2 mM thymidine, 2 h without thymidine, and 16 h with 100 nM nocodazole. The mitotically arrested cells were collected by shake-off, lysed with SDS sample buffer and processed for immunoblotting. For immunostainings, cells were grown on poly-L-lysine coated glass coverslips, fixed with 4% paraformaldehyde, permeabilized with 0.5% Triton X-100, blocked in 1% BSA/PBS, and incubated at room temperature with the primary and secondary antibodies for 60 and 45 min, respectively. DNA was stained with DAPI for 15 min at room temperature. The coverslips were mounted with Mowiol before microscopic observation.

Immunofluorescence with biotinylated peptide. The biotinylated peptides were detected with Streptavidin-Alexa Fluor®594 conjugate (Invitrogen). The immunostaining was done as described by Aubry et al[7]. In brief, biotin-labeled peptides were incubated with mitotically arrested U2OS cells in 200 μl DMEM for 3 h at 37°C. Cells were washed 3 times with cold medium and incubated with unlabeled avidin (50 μl; 10 μM) for 5 min at 37°C before fixation in 4% paraformaldehyde (37°C, 15 min), and permeability was induced with 0.1% Triton X-100 in PBS (RT, 5 min). Fixed cells were then blocked in PBS with 10% FCS (RT, 2 h) and incubated with H3T3ph antibody in PBS (RT, 2h), washed 2 times with PBD and finally with Alexa-594-Streptavidin and Alexa-633 secondary antibody (RT, 1h) in PBS. Before imaging the nucleus was stained with Hoechst 33342 in PBS (RT, 10 min).

Immunofluorescence. Images with single optical section were acquired at 21°C with a Zeiss 510 META laser-scanning confocal microscope equipped with a Plan Apochromat® 63×1.40 NA oil DIC objective. Images were deconvoluted with Zeiss 510 image software. Final images were processed and assembled using Photoshop® CS3 (Adobe). Brightness and contrast were adjusted using only linear operations applied to the entire image. The quantification data are plotted with Origin 8.1 software (OriginLab software).

Cell viability assay. A total of 10,000 cells were incubated for 24 hours with different peptide concentrations. Peptide cytotoxicity in adherent cell lines was analyzed by a colorimetric assay using 3-(4,5-dimethylthiazol-2-yl)-2, 5-diphenyltetrazolium bromide (called MTT) for adherent cells as described by the manufacturer (Sigma). In brief, U2OS cells were plated in a 24 well plate, and different concentrations of **PDP3** and **PDP3m** dissolved in 200 μl DMEM were added to cells and incubated for the different time points at 37°C. Then 20 μl of MTT (5mg/ml in PBS) was added to each well, shaken well and the cells were further incubated for 4 h at 37°C. Finally, the media is removed from each well and 200

μl of DMSO was added to thoroughly dissolve the purple formazan crystals. The solution is removed and the absorbance was measured at 670 nm.

Crystallization and structure determination. The gene coding for the α-isoform of PP1 (residues 1-330) fused with an N-terminal TEV-cleavable hexahistidine tag was synthesized (GenScript) and expressed under control of the Ptac promoter as described previously[8]. The protein was bound on TALON metal affinity resin (Clontech) and further purified on heparin sepharose and Superdex75 columns (GE Healthcare). The final purification and concentration steps were carried out in presence of the PDP2 peptide. The complex was concentrated to 6.5 mg/ml in a gel-filtration buffer (20 mM Tris (pH 7.5), 200 mM NaCl, 1 mM DTT) and subjected to crystallization by the sitting drop vapour diffusion method at 20ºC. The best crystals were obtained in 0.1 M Tris (pH 8.0), 1 M LiCl, 20% PEG6000. For data collection the crystals were transferred into the reservoir solution with 25% glycerol and flash frozen in liquid nitrogen.

X-ray diffraction data was collected at 1.1271 Å on the Proxima 1 beamline, Soleil, France, and processed using XDS[9]. The molecular replacement solution was found in Molrep[10] using 1FJM[11] as a search model. The structure was rebuilt in Coot[12] and refined in Refmac[9]. There are two copies of the PP1:**PDP2** complex in the asymmetric unit. The crystallographic statistics are presented in Supporting Table 3. The structure figures were prepared in Pymol (The PyMOL Molecular Graphics System, Schrödinger, LLC).

Supporting Table 3. Data collection and refinement statistics.*

Data collection	
Space group	$P4_22_12$
Cell dimensions (Å)	
a, b, c (Å)	138.0; 138.0; 113.7
α, β, γ (°)	90
Resolution range	98.0-3.16 (3.35-3.16)
R_{merge}	0.135 (0.581)
I/σ(I)	5.4 (1.3)
Completeness (%)	98.7 (99.5)
Redundancy	6.0 (5.9)
**Refinement**	
Resolution (Å)	98.0-3.10
No of reflections	21181
R_{work} / R_{free}	0.224 /0.279
No atoms	4946
B-factors	60.5
R.m.s. deviations	
Bond lengths (Å)	0.007
Angles (°)	1.017

*One crystal was used. Values in parentheses refer to the last resolution shell.

Supporting references

1. Beullens, M., Van Eynde, A., Stalmans W, Bollen M. The isolation of novel inhibitory polypeptides of protein phosphatase 1 from bovine thymus nuclei. *J. Biol. Chem.* **267**, 16538-16544 (1992).
2. Lesage, B., Beullens, M., Pedelini, L., Garcia-Gimeno, M.A., Waelkens, E., Sanz, P., Bollen, M. A complex of catalytically inactive protein phosphatase-1 sandwiched between Sds22 and inhibitor-3. *Biochemistry* **46**, 8909-8919 (2007).
3. Boudrez, A., Beullens, M., Waelkens, E., Stalmans, W., Bollen, M. Phosphorylation-dependent interaction between the splicing factors SAP155 and NIPP1. *J. Biol. Chem.* **277**, 31834-31841 (2002).
4. Lesage, B., Beullens, M., Nuytten, M., Van Eynde, A., Keppens, S., Himpens, B., Bollen, M. Interactor-mediated nuclear translocation and retention of protein phosphatase-1. *J. Biol. Chem.* **279**, 55978-55984 (2004).
5. Vander Mierde, D., Scheuner, D., Quintens, R., Patel, R., Song, B., Tsukamoto, K., Beullens, M., Kaufman, R.J., Bollen, M., Schuit, F.C. Glucose activates a protein phosphatase-1-mediated signaling pathway to enhance overall translation in pancreatic beta-cells. *Endocrinology* **148**, 609-617 (2007).
6. Qian, J., Lesage, B., Beullens, M., Van Eynde, A., Bollen, M. PP1/Repo-man dephosphorylates mitotic histone H3 at T3 and regulates chromosomal aurora B targeting. *Curr. Biol.* **21**, 766-773 (2011).
7. Aubry, S., Burlina, F., Dupont, E., Delaroche, D., Joliot, A., Lavielle, S., Chassaing, G., Sagan S. Cell-surface thiols affect cell entry of disulfide-conjugated peptides. *FASEB J.* **23**, 2956-2967 (2009).
8. Zhang, A.J., Bai, G., Deans-Zirattu, S., Browner, M.F., Lee, E.Y. Expression of the Catalytic Subunit of Phosphorylase Phosphatase (Protein Phosphatase-1) in *Escherichia coli. J. Biol. Chem.* **267(3)**, 1484-90 (1992).
9. Kabsch, W. XDS. *Acta Cryst.* D**66**, 125-132 (2010).
10. Collaborative Computational Project Number 4. The CCP4 suite: programs for protein crystallography *Acta Crystallogr.* D**50**, 760-763 (1994).
11. Goldberg J, Huang H.B., Kwon Y.G., Greengard P., Nairn A.C., Kuriyan J. Three-dimensional structure of the catalytic subunit of protein serine/threonine phosphatase-1. *Nature* **376**(6543):745-53 (1995)
12. Emsley P., Cowtan K. Coot: model-building tools for molecular graphics. Acta Crystallogr D**60**, 2126-2132 (2004).

Full reference [21] in the main manuscript: D. Huertas, M. Soler, J. Moreto, A. Villanueva, A. Martinez, A. Vidal, M. Charlton, D. Moffat, S. Patel, J. McDermott, J. Owen, D. Brotherton, D. Krige, S. Cuthill, M. Esteller, *Oncogene*. **2012**, *31*, 1408-1418.
